# Effects of Dietary Milk Thistle (*Silybum marianum*) Supplementation in Ducks Fed Mycotoxin-Contaminated Diets

**DOI:** 10.3390/vetsci10020100

**Published:** 2023-01-31

**Authors:** Jennifer Bencze-Nagy, Patrik Strifler, Boglárka Horváth, Nikoletta Such, Valéria Farkas, Károly Dublecz, László Pál

**Affiliations:** 1 Fiorács Ltd., Bonafarm Group, H-2941 Ács, Hungary; 2 Institute of Physiology and Nutrition, Georgikon Campus, Hungarian University of Agriculture and Life Sciences, H-8360 Keszthely, Hungary

**Keywords:** ducks, mycotoxins, deoxynivalenol, zearalenone, milk thistle, histopathology, liver, spleen, bursa of Fabricius

## Abstract

**Simple Summary:**

Previous experiments have shown that the medicinal plant milk thistle *(Silybum marianum)* can be a potential feed additive protecting farm animals against the deleterious effects of various mycotoxins. The main objective of our study was to investigate the health protective effects of dietary milk thistle seed (0.5%), oil (0.1%), and seed cake (0.5%) in ducks fed diets naturally contaminated with deoxynivalenol (3.43–3.72 mg/kg feed) and zearalenone (0.46–0.50 mg/kg feed). The feeding of experimental diets did not result in mortality cases, clinical signs of mycotoxicosis, or in differences of clinical chemistry values of blood serum but led to histopathological alterations. The positive effects of milk thistle oil on histopathology parameters exceeded those of the seed and seed cake (vacuolar hepatocyte degeneration) or all of the treatments showed similar effectiveness (solitary cell death and infiltration of lympho- and histiocytes in the liver, lymphocyte depletion in the spleen and bursa of Fabricius). Based on our results, the applied milk thistle supplements have been proven effective in the prevention of histopathological changes caused by deoxynivalenol and zearalenone.

**Abstract:**

The medicinal plant milk thistle (*Silybum marianum*) has been widely used due to its hepatoprotective properties. The main objective of our study was to investigate the health protective effects of dietary milk thistle seed (MS), oil (MO), and seed cake (MSC) in ducks fed diets naturally contaminated with deoxynivalenol (DON; 3.43–3.72 mg/kg feed) and zearalenone (ZEN; 0.46–0.50 mg/kg feed). Female White Hungarian ducks were randomly allocated to four dietary treatments consisting of the control diet (C), the control diet supplemented with 0.5% MS, 0.5% MSC, or 0.1% MO. The feeding of experimental diets did not result in mortality cases, clinical signs of mycotoxicosis, or in differences of clinical chemistry values of blood serum. The positive effect of MO on vacuolar hepatocyte degeneration exceeded that of the MSC on d14 and both MS and MSC on d42. Each treatment was equally effective in the decrease of the severity of solitary cell death and infiltration of lympho- and histiocytes in the liver on d28 as well as in the prevention of lymphocyte depletion in the spleen and bursa of Fabricius on d14. In conclusion, the applied treatments have been proven effective in the prevention of histopathological changes caused by DON and ZEN.

## 1. Introduction

Mediterranean plant, milk thistle (*Silybum marianum* L.) is mainly cultivated as a medicinal plant also in Hungary. The main active component of milk thistle seed is called silymarin. It is a complex which is composed of 70–80% of various flavonolignans (silybin, silydianin, silychristin, isosilybin, isosilychristin, dehydrosilybin, silandrin) and flavonoids (taxifolin, quercetin) and a mixture of polyphenol-like molecules (20–30%) that are not precisely defined chemically [1]. Silybin, which is present in the highest concentration in silymarin, is used in human and veterinary medicine primarily for the prevention and regeneration of liver diseases, due to its antioxidant [2,3] and antifibrotic effects [4] and partly due to its positive effect on protein synthesis [5]. Substances of the silymarin complex are well known for their immunostimulatory effect [6], but they also have excellent antibacterial [7] and antiviral [8] activity against some microorganisms. Milk thistle seeds also contain betaine and trimethyl glycine [9]. The ether extract of the seed oil is a rich source of vitamin E and polyunsaturated fatty acids, mainly linoleic and oleic acids, which are also involved in the hepatoprotective effects of milk thistle [10].

Cereals are the main ingredients of poultry diets and are rich sources of fusariotoxins. Deoxynivalenol (DON) was the most prevalent *Fusarium* mycotoxin followed by fumonisins and zearalenone (ZEN) detected in 64%, 60%, and 45% of all feed samples (74,821) from 100 countries monitored in a large-scale survey from 2008 to 2017 [11]. As in our study, the simultaneous presence of DON and ZEN is quite common and was observed in 39% of corn samples in the previous survey [11]. The high intake of corn during the force-feeding period of ducks can lead to high exposure of this poultry species to mycotoxins. DON binds to ribosomes and causes ribotoxic stress, which can lead to protein kinase activation, gene expression modulation, protein synthesis inhibition, and cell toxicity [12]. ZEN is similar to oestrogen hormones and able to bind to oestrogen receptors. Besides its provoked alterations in the reproductive system, it induces modifications in some hepatic enzyme function, and it can cause liver lesions. ZEN has been reported to damage liver and kidney tissue, to alter immune processes and to enhance lipid peroxidation [13]. Some experiments suggest that *Silybum marianum* can be successfully used as a feed additive protecting farm animals against the deleterious effects of various mycotoxins [14,15,16]. The active ingredients of milk thistle protect the liver and kidney from several exo- and endotoxins, which can be partly explained by the binding of silymarin to various toxins. Furthermore, silymarin can prevent the absorption of toxins into the hepatocytes by occupying the binding sites as well as by inhibiting many transport proteins at the cell membrane [1]. With an increase in membrane stability, it exerts a regulatory action on cellular and mitochondrial membrane permeability during xenobiotic injury [17]. The active ingredients of milk thistle participate in the neutralization of free radicals due to their antioxidant properties and they also support and regenerate the liver damaged by oxidative stress and mycotoxins [18]. Milk thistle also contributes to the control of toxicoses by increasing the excretion of toxic substances. Although some of the toxins are excreted in the bile, the other part of the toxins is reabsorbed in the body because the toxin-glucuronic acid bond is broken down by β-glucuronidase. However, silymarin reduces the activity of this enzyme, thereby promoting detoxification processes [19].

Previous studies with poultry species have shown the positive effects of silymarin or the whole seed of the plant *Silybum marianum* against mycotoxins. Silymarin as a complex of active ingredients derived from milk thistle was found as hepato- and nephroprotective against ochratoxin A intoxication in broiler chickens and laying hens [16,20]. The protective effects of a silymarin product was observed on the liver of broilers fed a diet contaminated with aflatoxin and fumonisin [21]. The hepatoprotective effect of ground milk thistle seed was demonstrated by measuring serum activities of aspartate-aminotransferase (AST), alanine aminotransferase (ALT) and alkaline phosphatase (ALP) in broiler chickens fed aflatoxin B1-contaminated diet [15]. In ducks, a more sensitive species to fusariotoxins than broilers and turkeys, the milk thistle seed supplementation had cytoprotective effects in the liver of animals fed diets contaminated with DON and ZEN [22].

The dietary milk thistle supplements, like the milk thistle seed, seed oil, and seed cake contain the potential active ingredients in different concentrations. Silymarin is concentrated mainly in the seed shell and the majority of this ingredient remains in the seed cake after oil extraction. The oil may contain some remnants of silymarin. However, its main health-protective ingredients are polyunsaturated fatty acids, tocopherols, and phytosterols, whose potential protective effects in mycotoxicosis have not been investigated in poultry yet. Therefore, the aim of our study was to investigate the effects of dietary milk thistle seed, oil, and seed cake in ducks fed diets contaminated with DON and ZEN.

## 2. Materials and Methods

### 2.1. Experimental Animals and Dietary Treatments

A total of 80, one-day-old female White Hungarian ducks were housed in deep litter floor pens and randomly divided into four treatment groups with five pens per treatment (n = 20/treatment, n = 4/pen). The animals did not receive any pharmacological treatments or vaccines during the experiment. Starter and grower diets were fed from day 0 to 14 and from day 15 to 42, respectively. The dietary treatments consisted of the control diet (C), the control diet supplemented on top with 0.5% milk thistle seed (*Silybum marianum*; MS), 0.5% milk thistle seed cake (MSC), or 0.1% milk thistle seed oil (MO). Milk thistle supplements were purchased from Safimpex Ltd. (Vác, Hungary). The corn used for the experimental diets was naturally contaminated with mycotoxins. The analyzed concentrations of mycotoxins in corn were 4.9 mg/kg deoxynivalenol (DON) and 0.66 mg/kg zearalenon (ZEN). The calculated concentration of DON was 3.43 and 3.72 mg/kg, while the calculated concentration of ZEN was 0.46 and 0.50 mg/kg in the starter and grower diets, respectively. The composition and calculated nutrient content of control diets can be seen in Table 1. Feed and water were provided ad libitum throughout the study. Chopped straw was used as bedding material. Room temperature, humidity, and ventilation rate were controlled according to the needs of the duck breed during the experiment.

### 2.2. Clinical Status, Measurement of Organ Weights, and Sampling

The clinical status of the ducks was observed daily and the signs of feed refusal, diarrhea, atypical behavior of birds (tonic immobility reaction or strutting behavior) and the presence of undigested feed in excreta were examined. On days 14, 28, and 42 five (d14 and 28) and eight (d42) ducks per treatment group were randomly selected, their individual body weight was measured, and blood samples of 1 mL per animal were collected from the wing vein (*v. cutanea ulnaris*). Serum was separated after centrifugation at 5500× *g* for 10 min, aspirated by pipette and transferred into 1.5 mL Eppendorf tubes and stored at −20 °C until further analysis. Selected ducks were terminally anaesthetized with carbon dioxide and the liver, spleen, and bursa of Fabricius were removed and weighed. The oropharyngeal cavity and esophagus of birds were inspected macroscopically. Relative organ weights were calculated as a percentage of the live body weight of animals measured before slaughter. For histological examination, samples of 10 g of the listed organs were fixed in a 10% buffered formaldehyde solution.

### 2.3. Analytical and Histopathological Examination Methods

Concentrations of DON and ZEN in corn were determined using the commercially available ELISA kits RIDASCREEN™ DON and RIDASCREEN™ ZEN (R-Biopharm GmbH, Darmstadt, Germany). The clinical chemical analyses of serum samples (AST and ALT activity, concentrations of glucose, cholesterol, triglyceride, creatinine, and uric acid) were performed by Vet-Med-Labor Ltd. using colorimetric assay kits (Diagnosticum Co., Budapest, Hungary) based on spectrophotometric methods. Histopathological examinations were performed by Autopsy KKT (Budapest, Hungary). The liver, spleen, and bursa of Fabricius samples in formaldehyde solution were embedded in paraffin and 5 μm thick sections were stained with hematoxylin and eosin. Tissue morphology was observed under a light microscope. The mean histological score was derived from the grade and stage of histological lesions seen in the investigated organs of the affected animals. The listed lesions were characterized per animal (1 point = mild, 2 points = medium, 3 points = high-grade alterations) and then mean score values were calculated in the group. The extent of vacuolar degeneration of hepatocytes, solitary hepatocyte necrosis, individual cell deaths of the mononuclear phagocyte system (MPS), focal lymphocytic and histiocytic interstitial infiltrates and interstitial fibrosis in liver samples, as well as lymphocyte counts in spleen and bursa of Fabricius samples, were evaluated.

### 2.4. Statistical Methods

Data of organ weights and serum clinical chemical parameters were evaluated by one-way analysis of variance after testing of normal distribution (Kolmogorov–Smirnov test) of data and homogeneity of variances (Levene-test). In the case of histopathological data, the Kruskal–Wallis analysis and Chi-Square test were used for the evaluation of the mean score and ratio of affected animals, respectively. Significance was determined at *p* ≤ 0.05. Statistical analysis was performed with SPSS 20.0 for Windows software package.

## 3. Results

### 3.1. Health Status and Relative Weight of Organs

No ducks died and no clinical signs of mycotoxicosis were observed during the experiment. Gross macroscopic inspection of oropharyngeal cavity and esophagus of ducks did not reveal signs of irritation, inflammation, or other pathological abnormalities. Effects of dietary treatments on the body weight of animals and the relative weight of liver, spleen, and bursa of Fabricius of ducks are presented in Table 2. Dietary supplementation of MO decreased the body weight of ducks compared to the control birds on day 42 significantly (*p* < 0.05). The relative liver weight of animals was not significantly influenced by milk thistle treatments. The relative spleen weight was lower in the MSC than in the C group and the relative bursa of Fabricius weight was lower in the MS than in the MO group on day 28 (*p* < 0.05).

### 3.2. Clinical Chemistry of Blood Serum

Among the measured serum chemical parameters, only serum creatinine concentration was significantly affected by dietary treatments (Table 3). Diets supplemented with MO increased serum creatinine levels of ducks compared to animals fed a control diet on days 28 and 42 (*p* ˂ 0.05).

### 3.3. Histopathology of Liver, Spleen, and Bursa of Fabricius

**Figure 1:**
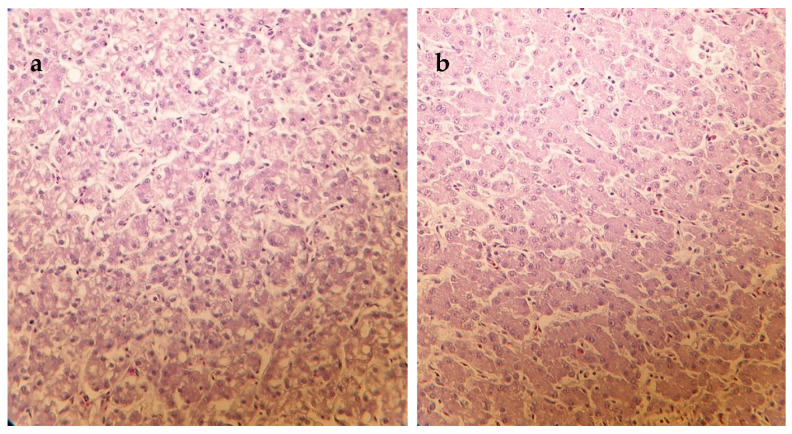
Hematoxylin and eosin-stained sections of duck’s livers (**a**) Liver section of a duck fed mycotoxin-contaminated control diet showing medium grade vacuolization of hepatocytes (day 14), 400× magnification; (**b**) Liver section of a duck fed mycotoxin contaminated diet supplemented with milk thistle seed showing healthy hepatocytes (day 14), 400× magnification.

**Figure 2:**
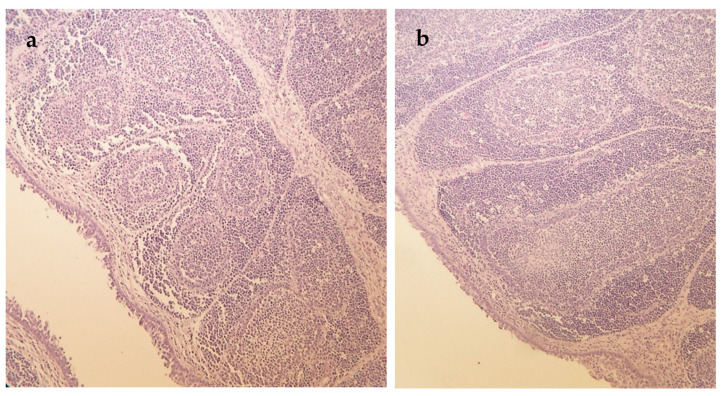
Hematoxylin and eosin-stained sections of the bursa of Fabricius of ducks (**a**) Bursa of Fabricius section of a duck fed mycotoxin contaminated control diet showing mildly decreased lymphocyte counts (day 14), 200× magnification; (**b**) Bursa of Fabricius section of a duck fed mycotoxin contaminated diet supplemented with milk thistle seed showing normal lymphocyte counts (day 14), 200× magnification.

The results of histological examinations are summarized in Table 4. Liver samples of ducks fed the C diet contaminated with mycotoxins showed medium-high grade vacuolar degeneration of hepatocytes on days 14 and 42 of the experiment (Figure 1). In the liver, this type of histopathological alteration due to the mycotoxin-contaminated diet was the most pronounced. Supplementation of MO and MS (day 14) and MO (day 42) to the feed decreased the cell degeneration significantly to a mild level according to mean score values compared to the C group (*p* < 0.05). Moreover, the ratio of affected animals was lower in the MO than in the C group on day 42 (*p* < 0.05). The feeding of mycotoxin contaminated C diet resulted in solitary hepatocyte necrosis of mild level on day 28 while there were no symptoms of cell necrosis in the MO and MS groups (*p* < 0.05). In addition, a lower ratio of animals also showed this sign in the MSC than in the C group. A mild-medium (day 28) and mild (day 42) grade of interstitial infiltration of lympho- and histiocytes as well as interstitial fibrosis were observed in the liver of control animals. The milk thistle treatments MSC and MS reduced the mean score values of interstitial infiltration, while each milk thistle diet decreased the ratio of animals affected by this alteration compared to the C group significantly (*p* < 0.05). Furthermore, the significant positive effect of milk thistle treatments could also be detected in the case of interstitial fibrosis on days 28 and 42 (*p* < 0.05). The mild-medium grade symptoms observed in each animal of the C group on day 28 could not be seen in ducks in the milk thistle treatment groups. The significant positive effect of MO in lowering the ratio of affected animals compared to the C group was shown on day 42 as well (*p* < 0.05). The mycotoxin-contaminated C diet resulted in mildly decreased lymphocyte counts of the spleen and bursa of Fabricius only on day 14 (Figure 2). These histological alterations did not occur at all in the groups where any kind of milk thistle supplementation was applied (*p* < 0.05).

## 4. Discussion

In our study, the potential health-protecting effect of milk thistle against mycotoxins was investigated in ducks fed corn-based diets contaminated with DON and ZEN. The three main possible feed supplements, milk thistle seed, seed cake, and seed oil, were compared in one experiment with poultry for the first time.

The sensitivity of ducks to various fusariotoxins may differ from that of broilers, layers, and turkeys. However, there are no different EU advisory limit concentrations or dietary guidance levels for fumonisins, T-2, DON, and ZEN for different poultry species. In our study, the measured dietary concentration of DON (4.9 mg/kg) and ZEN (0.66 mg/kg) in corn were lower than the maximum EU guidance values for cereals (DON: 8 mg/kg; ZEN: 2 mg/kg; Commission Recommendation 2016/1319/EC). The calculated dietary concentration of DON (3.43 and 3.72 mg/kg in the experimental starter and grower diets, respectively) in our experiment was also lower than the maximum guidance value in poultry complete diets (DON: 5.0 mg/kg; 2016/1319/EC). At the moment, there is no EU guidance level for ZEN in poultry compound feed. The calculated levels of ZEN were 0.46 and 0.50 mg/kg in the experimental starter and grower diets, respectively, and feeding diets with similar dietary concentrations of the toxin did not result in clinical signs of ducks [22,23].

In accordance with the dietary toxin concentrations, the feeding of diets contaminated with DON and ZEN did not result in mortality cases, clinical signs of mycotoxicosis, or macroscopic lesions of the intestinal tract in our study. The toxic load was well tolerated by the experimental animals and the body weight of animals was characteristic of the breed standard and also met the results of a previous study where the same White Hungarian duck breed was used in the experiment [22]. Furthermore, the relative weight of the liver, spleen, and bursa of Fabricius did not show the negative effects of dietary DON and ZEN, the results of organ weights were typical for healthy ducks at each sampling time [23,24]. According to previous studies, growing ducks like other poultry species are quite tolerant to dietary concentrations of DON up to 5.8–7 mg/kg and do not show adverse effects on health and performance [25,26], but differences in relative organ weights, e.g., decreased weight of bursa of Fabricius in a dose-related fashion, may occur [23,27]. As one possible reason for the low toxicity of DON for poultry is a pronounced renal first pass of this fusariotoxin [25,28]. Similarly to the low sensitivity to DON, poultry seems to be quite tolerant to ZEN, which could be explained by the high estrogen concentration in poultry blood [29]. No change in performance and organ weights of force-fed mule ducks exposed to ZEN at a concentration of 0.5 mg/kg feed were observed [23]. However, the same study revealed that combined exposure of ducks to fumonisin B1 and B2 (20 mg/kg), DON (5 mg/kg), and ZEN (0.5 mg/kg) can lead to decreased performance. The dietary milk thistle treatments influenced the body weight of ducks only at the end of the experiment when the body weight of animals in the MO group was significantly lower than in the C group. Dietary silymarin or milk thistle supplements can increase the body weight gain of ducks [24] and broilers fed diets without mycotoxin contaminations [14,27], but the negative effect of milk thistle seed products on the growth of broilers has also been demonstrated [30,31]. The positive effects of dietary milk thistle and silymarin supplementation on the growth performance of poultry fed mycotoxin-contaminated diets were reported in experiments only with broilers and focusing on aflatoxin B_1_ or combined treatments of aflatoxins and other mycotoxins [15,32]. Studies investigating the potential performance protective effect of silymarin in ducks showing the adverse effect of dietary DON and ZEN are lacking. In our study, the significant differences in the relative weight of the spleen and bursa of Fabricius among treatment groups were observed temporarily only on day 28. Silymarin supplementation did not affect the relative liver and lymphoid organ weight of growing ducklings [24], and the reasons for these effects of milk thistle treatments on organ weights in the present experiment are obscure.

The serum concentrations of some biochemical parameters associated with hepatic and renal health were in the physiological range, indicating that the mycotoxin contamination of experimental diets did not cause severe injuries to the liver and kidneys. In agreement with our results, the feeding of DON-contaminated wheat (5.8 mg/kg) to one-year-old captive mallards did not lead to changes in serum chemistry [26]. Similarly, feeding diets containing the maximum EU tolerated levels of DON (5 mg/kg) and ZEN (0.5 mg/kg) alone or in combination did not affect the concentrations of proteins, cholesterol, uric acid, and activity of lactate dehydrogenase (LDH), alkaline phosphatase (ALP) and ALT in the blood plasma of 84-day-old ducks [23]. In our study, the serum creatinine concentration in the control group was significantly lower than in the milk thistle seed oil treatment group on days 28 and 42 and similar tendencies were observed for the two other milk thistle treatment groups. Creatinine is a byproduct of muscle energy metabolism and is used for the determination of kidney dysfunction and muscle disorders [33]. In the case of elevated plasma levels, the avian renal tubules may secrete creatinine and reabsorb creatinine when plasma levels are normal, so the use of this parameter as the only indicator value for kidney health might be misleading in birds [33]. Silymarin could be protective against renal toxicity by maintaining the serum creatinine level on the physiological level according to studies with mammals [34]. However, the effects of either dietary silymarin or milk thistle on serum creatinine in poultry mycotoxin studies with DON and ZEN have not been investigated yet. The fusariotoxins DON and ZEN can increase serum creatinine concentration through damage in kidney function in mice [35], but other authors reported that creatinine levels were decreased by ZEN [36]. The damaging effects of DON and ZEN on kidney function are often a result of the induced oxidative stress based on the accumulation of reactive oxygen species (ROS) and reduced activity of antioxidant enzymes, such as superoxide dismutase (SOD) [35]. This type of effect of ZEN causing lower serum creatinine levels together with the protective role of milk thistle in our study cannot be excluded, but further studies are needed to verify this assumption in ducks.

The liver, spleen, and bursa of Fabricius showed histopathological alteration due to the DON and ZEN contaminated diets in the present experiment. The nature and extent of the majority of observed lesions could be classified as mild, basically not influencing the function of the affected organ and prone to regeneration. The feeding of diets contaminated with DON alone or together with ZEN can provoke vacuolar degeneration of hepatocytes, individual necrosis of the mononuclear phagocyte system, focal lymphocytic and histiocytic interstitial infiltration, and interstitial fibrosis [22,23]. In our study, the most pronounced pathological alteration in the liver was the vacuolar degeneration of the hepatocyte cytoplasm, which is usually related to a disturbance in the hydration status or lipid metabolism, and mostly indicates an increased metabolic and/or detoxification activity of the cell. The accumulation of lipids in the cytoplasm of hepatocytes may be a protective physiological process against lipotoxicity and essential for normal liver regeneration [22]. Milk thistle treatments used in our study were effective in reducing the severity and/or the ratio of affected animals associated with the observed histopathological signs of the duck liver. A similar positive effect of dietary 0.5% MS on vacuolar degeneration of hepatocytes in ducks fed DON- and ZEN-contaminated diets was found [22]. The authors concluded that the exposure of the two fusariotoxins caused oxidative stress and the milk thistle supplementation was able to normalize and further enhance the antioxidant defense system. Previous studies reported that silymarin as a natural antioxidant molecule can trigger various antioxidant enzymes and stimulate non-enzymatic nuclear factor erythroid 2-related factor 2 (Nrf2) pathways, which consequently diminishes oxidative stress [37]. Parameters of the antioxidant defense system are not measured in our study. However, the protective antioxidant effect of silymarin could be a key factor in the milder histological changes in the milk thistle treatment groups. Interstitial fibrosis and interstitial infiltration of lympho- and histiocytes can usually occur as a result of liver cell tissue damage and repair during poultry fattening and the mild form is asymptomatic [23]. Silymarin and its bioactive constituents are able to hinder the progression of initial liver fibrosis and has been proven as hepatoprotective [38]. Fibrosis and inflammation produce fibrous scarring through the activation of myofibroblasts in the liver, which consequently exudes extracellular matrix proteins. Furthermore, the augmentation of hepatic stellate cells (HSCs) and Kupffer cells is important in the production of hepatic fibrosis [39]. Experimental investigations have shown that silymarin impairs the proliferation of HSCs and prevents their translation into myofibroblasts, while also down-regulating gene expression of the extracellular matrix components required during fibrosis [40].

A decrease in the number of lymphocytes in the Malpighian bodies of the spleen and the follicles of the bursa of Fabricius (lymphocyte depletion) usually occurs as a result of toxic or various stress effects. The immune organs are considered as target organs of fusariotoxins and histological findings can reveal lymphocyte depletion in spleen and bursa of Fabricius of animals fed diets contaminated with DON and ZEN [23,41,42]. The mild form of lymphocyte depletion was seen only on day 14 in animals without milk thistle treatment and was regenerated. The cells are replaced and resettled through division by day 28. The similar protective effects of silymarin was observed only in one mycotoxin study where the depletion of lymphoid cells in the bursa of Fabricius and spleen in broilers fed ochratoxin A contaminated diet were reported and the silymarin supplementation was proved to be preventive in the development of the symptoms [16].

As a novelty of our study, the findings of the histopathological examinations allowed us to compare the effectiveness of different milk thistle treatments within one experiment. Previous studies have showed the hepatoprotective effects of MSC and MO [30,43], but our experiment is the first where these treatments proved their positive effects in ducks exposed to DON and ZEN. All three milk thistle treatments used, the seed, the seed cake, and the seed oil, were equally effective in the decrease of the severity and/or the ratio of affected animals in the case of solitary cell death and infiltration of lympho- and histiocytes in the liver on day 28. Moreover, each treatment showed similar effectiveness in the prevention of lymphocyte depletion in the spleen and bursa of Fabricius. As for vacuolar hepatocyte degeneration, the positive effect of MO exceeded that of the MSC on day 14 and both MS and MSC at the end of the experiment. Furthermore, the MO was the only treatment whose significant positive effect in preventing liver interstitial fibrosis remained until the end of the experiment.

The health-protective ingredients potentially responsible for the observed effects in our study were in different concentrations in the treatments. The seeds of *Silybum marianum* contain a relatively high amount of oil (20–25%) rich in polyunsaturated fatty acids, tocopherols, and phytosterols. The oil has to be removed from the seeds before the extraction of the silymarin complex of flavonolignans and flavonoids [1]. The silymarin concentration of MS ranges from 10 to 30 g/kg for different genotypes and in different environmental conditions [44]. The MO and MSC fractions are obtained by cold pressing the seed. There could be some remnants of silymarin in MO after pressing (130–190 µg/mL, personal communication) and the MSC contains 15–40 g/kg silymarin which is similar to the concentrations measured in the seed [45,46]. Besides silymarin components, the phytochemicals found in MO, such as tocopherols, phytosterols, and ascorbic acid 2,6 dihexadecanoate, can serve as potent antioxidants [43,47,48]. Antioxidant activity is one of the main effects responsible for the hepatoprotective nature of MS and both MO and MSC were also shown as feed and food supplements with antioxidant properties [43,48].

## 5. Conclusions

In conclusion, the DON and ZEN contamination of diets caused only subclinical toxicosis in our experiment, and the experimental animals generally successfully tolerated the toxic load. The applied mycotoxins did not change the serum biochemistry parameters significantly. However, the liver, spleen, and bursa of Fabricius showed histopathological alteration due to the mycotoxin-contaminated diets. The applied three milk thistle products, the seed, the seed cake, and the seed oil, have been proven effective based on the mean histological score and the ratio of affected animals in the prevention of histopathological changes observed and could be used as potent feed supplements for ducks.

## Figures and Tables

**Table 1:** Dietary composition and calculated nutrient content of control diets.

Ingredients (g/kg)	Starter Diet (d0–14)	Grower Diet (d15–42)
Corn	700.0	760.0
Extracted soybean meal	266.0	210.0
Calcium carbonate	12.0	11.0
Monocalcium phosphate	12.0	10.0
Sodium chloride	4.0	4.0
DL-Methionine	1.0	0.0
Premix ^1^	5.0	5.0
**Calculated nutrient content (g/kg)**		
AME_n_ (MJ/kg)	12.31	12.62
Dry matter	887.3	885.9
Crude protein	185.4	165.0
Crude fat	30.7	31.9
Crude fiber	27.9	26.0
Calcium	8.6	7.7
Available phosphorous	3.5	3.0
Lysine	9.5	8.0
Methionine	5.0	3.8
Methionine + cysteine	8.2	6.7

^1^ The active ingredients of the premixes were as follows (per kg of diet): Starter premix:—vitamin A—14,000 IU, vitamin D_3_—3600 IU, vitamin E—54 IU, vitamin K_3_—5.85 mg, thiamine—3.24 mg, riboflavin—11.8 mg, pyridoxine HCl—6.3 mg, cobalamin—0.03 mg, niacin—58.5 mg, pantothenic acid—15.2 mg, folic acid—2.25 mg, biotin—0.32 mg, betaine—180 mg, choline-chloride—270 mg, Zn—90 mg, Cu—13.5 mg, Fe—54 mg, Mn—90 mg, I—2.7 mg, Se —0.36 mg; Grower premix:—vitamin A—12,000 IU, vitamin D_3_—3200 IU, vitamin E—48 IU, vitamin K_3_—5.2 mg, thiamine—2.88 mg, riboflavin—10.56 mg, pyridoxine HCl—5.6 mg, cobalamin—0.03 mg, niacin—52 mg, pantothenic acid—13.52 mg, folic acid—2.0 mg, biotin—0.28 mg, betaine—160 mg, choline-cloride—240 mg, Zn—80 mg, Cu—12 mg, Fe—48 mg, Mn—80 mg, I—2.4 mg, Se—0.32 mg.

**Table 2:** Effect of dietary treatments on the body weight and the relative weight^1^ of liver, spleen, bursa of Fabricius of ducks.

Variables	Group	Day 14		Day 28		Day 42	
		Mean	SEM	Mean	SEM	Mean	SEM
Body weight (g)	C	313.60	15.75	1160.40	66.21	2393.00 ^a^	80.23
	MO	307.60	23.18	1184.80	38.40	2102.75 ^b^	42.53
	MSC	268.40	23.99	1090.40	40.53	2262.00 ^ab^	52.54
	MS	355.20	32.61	1216.00	29.83	2152.50 ^ab^	52.42
*Significance*		NS		NS		*p* < 0.05	
Liver relative weight (%)	C	4.55	0.42	2.73	0.20	2.72	0.06
	MO	4.15	0.07	2.79	0.15	2.34	0.09
	MSC	4.55	0.32	2.54	0.11	2.54	0.09
	MS	4.12	0.22	2.74	0.11	2.67	0.16
*Significance*		NS		NS		NS	
Spleen relative weight (%)	C	0.09	0.01	0.10 ^a^	0.01	0.06	0.01
	MO	0.10	0.02	0.07 ^ab^	0.01	0.07	0.01
	MSC	0.09	0.02	0.06 ^b^	0.01	0.07	0.01
	MS	0.11	0.00	0.08 ^ab^	0.01	0.07	0.01
*Significance*		NS		*p* < 0.05		NS	
Bursa of Fabricius relative weight (%)	C	0.18	0.03	0.13 ^ab^	0.01	0.11	0.01
	MO	0.19	0.02	0.14 ^a^	0.01	0.11	0.01
	MSC	0.19	0.04	0.11 ^ab^	0.01	0.12	0.01
	MS	0.22	0.03	0.09 ^b^	0.01	0.11	0.01
*Significance*		NS		*p* < 0.05		NS	

C: control, MO: milk thistle oil, MSC: milk thistle seed cake, MS: milk thistle seed; NS: non-significant (*p* > 0.05). ^1^ Weight of organs in relation to body weight; ^ab^ Means in the same column with different superscripts are significantly different (*p* < 0.05).

**Table 3:** Effect of dietary treatments on the blood serum concentrations of some chemical parameters.

Variables	Group	Day 14		Day 28		Day 42	
		Mean	SEM	Mean	SEM	Mean	SEM
AST (IU/L)	C	56.20	2.37	135.00	18.76	13.83	0.95
	MO	69.80	13.00	110.80	13.75	16.13	1.08
	MSC	58.40	7.80	131.40	30.00	13.63	1.10
	MS	64.20	5.10	137.60	30.56	12.14	1.67
*Significance*		NS		NS		NS	
ALT (IU/L)	C	50.00	1.52	38.20	3.38	26.50	1.82
	MO	52.80	7.44	34.40	3.18	27.25	2.19
	MSC	54.20	2.73	42.20	3.18	28.88	2.22
	MS	53.20	2.63	39.40	1.81	25.43	1.04
*Significance*		NS		NS		NS	
Glucose (mmol/L)	C	9.18	0.35	9.02	0.16	8.20	0.45
	MO	8.98	0.53	8.72	0.22	7.89	0.31
	MSC	8.96	0.38	8.72	0.39	7.48	0.21
	MS	8.72	0.56	8.96	0.29	7.84	0.15
*Significance*		NS		NS		NS	
Cholesterol (mmol/L)	C	4.48	0.13	5.40	0.37	4.38	0.25
	MO	4.50	0.21	5.36	0.34	3.84	0.11
	MSC	4.54	0.10	5.24	0.26	4.23	0.22
	MS	4.36	0.27	5.18	0.51	5.14	0.66
*Significance*		NS		NS		NS	
Triglycerides (mmol/L)	C	1.73	0.27	0.99	0.13	1.66	0.28
	MO	1.46	0.27	0.89	0.07	1.75	0.39
	MSC	1.89 1.20	0.28	1.08	0.14	1.18	0.14
	MS	1.20	0.22	1.06	0.11	1.01	0.16
*Significance*		NS		NS		NS	
Creatinine (µmol/L)	C	21.00	0.71	19.00 ^b^	0.45	18.50 ^b^	0.81
	MO	22.40	0.68	23.20 ^a^	1.39	23.00 ^a^	1.00
	MSC	21.40	0.68	21.20 ^ab^	0.07	21.88 ^ab^	1.03
	MS	21.80	1.62	20.80 ^ab^	0.73	21.57 ^ab^	1.17
*Significance*		NS		*p* < 0.05		*p* < 0.05	
Uric acid (µmol/L)	C	244.40	51.52	114.40	11.89	196.67	30.60
	MO	292.40	74.67	142.20	17.25	174.13	21.70
	MSC	206.20	17.35	181.00	15.54	150.13	9.99
	MS	260.40	18.87	154.00	16.60	143.00	7.84
*Significance*		NS		NS		NS	

C: control, MO: milk thistle oil, MSC: milk thistle seed cake, MS: milk thistle seed; NS: non-significant (*p* > 0.05). ^ab^ Means in the same column with different superscripts are significantly different (*p* < 0.05).

**Table 4:** Effect of dietary treatments on histopathology of liver, spleen, and bursa of Fabricius of ducks.

Variables	Group	Day 14		Day 28		Day 42	
		Mean Score	Animals (%)	Mean Score	Animals (%)	Mean Score	Animals (%)
Liver							
vacuolar cell degeneration	C	2.6 ^a^	100.0	1.2	40.0	2.4 ^a^	100.0 ^a^
	MO	1.0 ^b^	60.0	0.4	40.0	0.6 ^b^	50.0 ^b^
	MSC	2.0 ^ab^	100.0	1.0	80.0	1.4 ^ab^	62.5 ^a^
	MS	1.0 ^b^	60.0	0.6	40.0	1.6 ^ab^	100.0 ^a^
*Significance*		*p* < 0.05	NS	NS	NS	*p* < 0.05	*p* < 0.05
solitary cell death	C	0.0	0.0	1.0 ^a^	100.0 ^a^	0.1	12.5
	MO	0.0	0.0	0.0 ^b^	0.0 ^b^	0.0	0.0
	MSC	0.0	0.0	0.4 ^ab^	40.0 ^b^	0.0	0.0
	MS	0.0	0.0	0.0 ^b^	0.0 ^b^	0.0	0.0
*Significance*		NS	NS	*p* < 0.05	*p* < 0.05	NS	NS
cell death of mononuclear phagocyte system	C	0.0	0.0	0.4	40.0	0.1	12.5
	MO	0.0	0.0	0.0	0.0	0.0	0.0
	MSC	0.0	0.0	0.2	20.0	0.0	0.0
	MS	0.0	0.0	0.0	0.0	0.0	0.0
*Significance*		NS	NS	NS	NS	NS	NS
interstitial infiltration of lympho- and histiocytes	C	0.0	0.0	1.6 ^a^	80.0 ^a^	1.1	87.5
	MO	0.2	20.0	0.4 ^ab^	40.0 ^b^	1.1	87.5
	MSC	0.0	0.0	0.2 ^b^	20.0 ^b^	0.9	62.5
	MS	0.2	20.0	0.0 ^b^	0.0 ^b^	0.9	75.0
*Significance*		NS	NS	*p* < 0.05	*p* < 0.05	NS	NS
interstitial fibrosis	C	0.0	0.0	1.6 ^a^	100.0 ^a^	1.1	87.5 ^a^
	MO	0.0	0.0	0.0 ^b^	0.0 ^b^	0.3	12.5 ^b^
	MSC	0.0	0.0	0.0 ^b^	0.0 ^b^	0.9	62.5 ^a^
	MS	0.0	0.0	0.0 ^b^	0.0 ^b^	0.6	50.0 ^a^
*Significance*		NS	NS	*p* < 0.05	*p* < 0.05	NS	*p* < 0.05
Spleen							
decreased lymphocyte count	C	1.0 ^a^	100.0 ^a^	0.0	0.0	0.0	0.0
	MO	0.0 ^b^	0.0 ^b^	0.0	0.0	0.0	0.0
	MSC	0.0 ^b^	0.0 ^b^	0.0	0.0	0.0	0.0
	MS	0.0 ^b^	0.0 ^b^	0.0	0.0	0.0	0.0
*Significance*		*p* < 0.05	*p* < 0.05	NS	NS	NS	NS
Bursa of Fabricius							
decreased lymphocyte count	C	1.0 ^a^	100.0 ^a^	0.0	0.0	0.0	0.0
	MO	0.0 ^b^	0.0 ^b^	0.0	0.0	0.0	0.0
	MSC	0.0 ^b^	0.0 ^b^	0.0	0.0	0.0	0.0
	MS	0.0 ^b^	0.0 ^b^	0.0	0.0	0.0	0.0
*Significance*		*p* < 0.05	*p* < 0.05	NS	NS	NS	NS

C: control, MO: milk thistle oil, MSC: milk thistle seed cake, MS: milk thistle seed; NS: non-significant (*p* > 0.05). ^ab^ Means in the same column with different superscripts are significantly different (*p* < 0.05).

## Data Availability

Not applicable.
